# Arginine supplementation improves lactate dehydrogenase levels in steady-state sickle cell patients: preliminary findings from Kinshasa, the Democratic Republic of Congo

**DOI:** 10.3389/fpain.2024.1391666

**Published:** 2024-11-22

**Authors:** Ange C. M. Ngonde, Philippe N. Lukanu, Ange Mubiala, Michel N. Aloni

**Affiliations:** ^1^ Polyclinique de Kinshasa, Kinshasa, The Democratic Republic of Congo; ^2^ Department de Médecine de Famille et Soins de Santé Primaires, Université Protestante du Congo, Kinshasa, The Democratic Republic of Congo; ^3^ Institut National de Recherche Biomédicale (INRB), Kinshasa, The Democratic Republic of Congo; ^4^ Département de Pédiatrie, Cliniques Universitaires de Kinshasa, Faculté de Médecine, Université de Kinshasa (UNIKIN), Kinshasa, The Democratic Republic of Congo

**Keywords:** sickle cell disease, L-arginine, hydroxyurea, LDH, Democratic Republic of Congo (DRC)

## Abstract

**Background:**

Sickle cell disease (SCD) disrupts oxygen transport due to the abnormal shape and rigidity of red blood cells, leading to hemolysis. Hemolysis, a major co-morbidity in SCD, is indicated by elevated levels of lactate dehydrogenase (LDH). Arginine depletion, which is essential for nitric oxide (NO) synthesis, contributes to various complications in SCD. L-arginine supplementation may increase NO levels and reduce oxidative stress. Research on its benefits in SCD, which is prevalent in sub-Saharan Africa, is limited. This study evaluates the effect of arginine supplementation on LDH levels in patients with steady state SCD.

**Methods:**

In a retrospective study, we evaluated the effect of arginine supplementation on LDH levels in a cohort of 31 patients. We divided the study into three phases: pre-HU treatment, HU treatment, and combined HU and arginine supplementation.

**Results:**

The cohort had a median age of 12 years, ranging from 2 to 43 years. Throughout all three phases of the study, lactate dehydrogenase (LDH) levels were consistently above the established normal ranges, with elevations of 216.7%, 220.3% and 176.6% above the normative values for baseline, Phase 1 (HU) and Phase 2 (HU + Arg), respectively. Specifically, LDH levels were 649.7 ± 364.2 U/L in Baseline Phase, 661.6 ± 367 U/L in Phase 1, and 529.9 ± 346.3 U/L in Phase 2. When comparing these discrete study intervals, it is noteworthy that LDH levels were significantly lower in Phase 2 compared to the previous phases (*p* = 0.002).

**Conclusion:**

Preliminary findings revealed a significant lower LDH levels among sickle cell patients receiving combined arginine supplementation and hydroxyurea (HU). Although these findings are promising, their credibility and applicability require further and more extensive research.

## 1 Introduction

Sickle cell disease (SCD) is a hereditary genetic disorder characterized by a complex interaction between vaso-occlusion and hemolysis, resulting in widespread systemic manifestations. Vaso-occlusion, characterized by the occlusion of small blood vessels, by rigid sickled red blood cells, constitutes the hallmark pathology underlying both acute and chronic complications associated with SCD. This phenomenon leads to a cascade of events causing tissue ischemia, inflammation, and organ damage. Concurrently, hemolysis, which is the premature destruction of red blood cells (RBCs), further complicates the clinical landscape by contributing to anemia, increased oxidative stress, and impairment of endothelial dysfunction (1).

The multifaceted pathophysiology of SCD is a complex web of molecular and cellular mechanisms that impact both vascular and hematological systems. At the core of this pathology is the presence of hemoglobin S (Hb S), an inherently unstable variant of hemoglobin that exhibits a propensity to polymerize in an oxygen-depleted environment. This polymerization serves as the initiator of the characteristic sickling exhibited by RBCs, ultimately leading to their impaired deformability and increased susceptibility to hemolysis, which, in turn, initiates the release of hemoglobin and its associated toxic catabolic by-products into the circulation (2).

Nitric oxide (NO), a potent vasodilator molecule, plays a central role in the complex regulation of vascular tone and endothelial function. It is synthesized by the enzymatic conversion of the amino acid L-Arginine by the enzyme nitric oxide synthase (NOS) in the endothelial cells. However, in the context of SCD, a disease characterized by its complexity, a state of NO resistance and decreased NO bioavailability emerges as a central phenomenon, primarily due to scavenging by free hemoglobin released during hemolysis, concomitant with increased oxidative stress (3). The situation is further exacerbated by reduced substrate availability, driven by the enzymatic conversion of L-arginine to ornithine and urea. This conversion is catalyzed by arginase, which is itself released from hemolyzed RBCs (4, 5). This phenomenon is the basis of certain complications such as pulmonary arterial hypertension in sickle cell patients.

The complex interplay between NO and the pathophysiology of SCD has prompted research efforts focused on interventions targeting the L-arginine-NO pathway. One such intervention is hydroxyurea (HU), a well-established disease-modifying therapy for SCD (6). HU has been shown to increase NO bioavailability, thereby mitigating endothelial dysfunction, and limiting vaso-occlusive complications. The therapeutic reach of HU also extends to increasing fetal hemoglobin (Hb F) levels. These multiple effects, in conjunction with its other mechanisms of action, contributes to a comprehensive mitigation of SCD-related complications (7, 8).

In addition to HU, supplemental administration of L-arginine has shown promise in increasing NO levels, potentially alleviating vaso-occlusive episodes. Investigations have shed light on the potential therapeutic benefits of arginine supplementation, particularly in improving pain management and mitigating acute complications in SCD patients (8–10).

It is worth noting that the existing body of research, particularly in resource-limited regions such as the Democratic Republic of Congo (DRC), underscore the need for comprehensive investigations aimed at elucidating the therapeutic potential of arginine supplementation. Of particular interest is its effect on lactate dehydrogenase (LDH) levels, recognized as a relevant marker of intravascular hemolysis (10, 11).

LDH plays a central role in cellular metabolism, catalyzing the conversion of pyruvate to lactate while facilitating the conversion of nicotinamide adenine dinucleotide (NADH) to its oxidized form, NAD. This ubiquitous enzyme is distributed in all tissues and is a diagnostic indicator of hemolysis in conjunction with parameters such as reticulocyte count, indirect bilirubin level, and serum haptoglobin. Notably, individuals with sickle cell disease often have elevated serum LDH levels, even during steady states (12). In addition, LDH has been shown to be a reliable biomarker of hemoglobinemia, NO consumption, and NO resistance in patients with sickle cell disease (13).

Given the significant socioeconomic burden of SCD, particularly in countries with high prevalence rates such as the DRC (14, 15), it is imperative to rigorouslyinvestigate the influence of arginine supplementation on LDH levels; as in Tanzania, where daily supplementation with arginine, citrulline and chloroquine has been shown to increase arginine bioavailability and as well as the measurement of nitric oxide-dependent endothelial function (16). Thisendeavor has considerable clinical and translational significance, offering potential insights intocomplex interplay between L-arginine availability, NO bioavailability, and LDH levels in the context of SCD. Such insights may pave the way for novel therapeutic strategies aimed at alleviating hemolysis-related complications and ultimately improving patient outcomes.

While L-carnitine has been shown to increase membrane stability in mature erythrocytes at physiological concentrations (17), it is important to note that L-carnitine administration alone does not affect pretreatment lactate dehydrogenase (LDH) levels (18). Among the components of the administered product, only arginine, which serves as a substrate for nitric oxide (NO) (8, 10), has a significant interaction with LDH. This interaction is significant because LDH serves as a biomarker for both hemoglobinuria and NO consumption and resistance in sickle cell patients (13). Consequently, L-carnitine is not the primary focus of this study.

This study addressed the following research question: In individuals with homozygous sickle cell disease in steady state, does the oral administration of a powdered supplement containing 500 mg of arginine (Arg) in addition to HU result in a significant modulation of LDH levels?

## 2 Methodology

### 2.1 Study design

A longitudinal interventional study covering the period from 2015 to 2019 was methodically conducted. This study was carried out within the framework of the Kinshasa Polyclinic, where patients were carefully followed and monitored.

### 2.2 Participants

The cohort studied consisted of 31 individuals diagnosed with sickle cell disease. The patients received continuous care in a day hospital at Kinshasa Polyclinic during the period from 2015 to 2019. The study framework was designed to include three distinct phases of medical surveillance, each with its own focus. These phases included: the baseline phase (19), prior to initiation of HU treatment, the intermediate phase, characterized by the administration of HU for a minimum of three months, and the subsequent phase, during which arginine supplementation was used concurrently with HU treatment for a minimum of three months (Supplementary Schema S1). The LDH level was considered to be in steady state (19), defined as a period of at least four weeks without crisis prior to the consultation and sample collection.

Participation of human research subjects conformed to institutional review board guidelines, applicable laws, and the World Medical Association Declaration of Helsinki.

### 2.3 Variables, measurements, and data collection

Quantification of LDH levels was performed using the Cobas c 111 automated analyzer. This automated system adheres to established reference ranges, standardized at a temperature of 37°C as follows: for women: 135–214 U/L; for men: 135–225 U/L; for children (2–15 years of age): 120–300 U/L, and for newborns (4–20 days of age): 225–600 U/L. Concomitant hematological assessments, including blood counts, were performed using the Sysmex XP-300 Automated Hematology Analyzer.

Hydroxyurea (HU) was administered within a fixed dose range of 20–30 mg/kg, with treatment regimens extending for a minimum of 3 months. This regimen was administered both as an isolated intervention and in conjunction with L-arginine (L-Arg) supplementation.

The L-arginine component, at a dose of 500 mg, was encapsulated in a trace element complex known as Fertilo forte. This complex included additional ingredients, specifically vitamin E (15 mg), folic acid (400 µg), zinc (2.25 mg), selenium (30 µg), L-carnitine (440 mg), and coenzyme Q10 (15 mg). The formulation was presented in a powdered stick format, with the prescribed dosage set at 1 stick per day for individuals 1–14 years of age and 2 sticks per day for those 15 years of age and older.

The dataset reviewed in this study was derived from the comprehensive medical records of outpatients diagnosed with SCD. The scope of data collection included a spectrum of variables, including age, sex, weight, LDH levels, and a comprehensive set of hematological parameters, including hemoglobin (Hb), hematocrit (Hct), white blood cell count (WBC), mean corpuscular volume (MCV), and mean corpuscular hemoglobin concentration (MCHC). Thus, only monthly LDH and blood count values recorded during a stable period of at least three months were considered for each of the three observation phases: the baseline phase, the phase during hydroxyurea (HU) treatment, and the phase of arginine (ARG) supplementation alongside HU therapy.

The inclusion and exclusion criteria are presented in Supplementary Table S1.

### 2.4 Statistical analysis

The LDH distribution was not Gaussian and this was confirmed by the Shapiro Wilk test. Then for this study, the Friedman test was used to perform a comparative analysis of LDH levels within the context of 3 groups observed at three different time points. Pairwise comparisons of LDH levels were performed using the Wilcoxon test signed-rank test. This comparison included baseline LDH vs. phase 1 LDH, baseline LDH vs. phase 1 LDH, and phase 1 LDH vs. phase 2 LDH.

The Spearman rank correlation test was used to assess the degree of association between LDH levels and hemoglobin levels, as well as between LDH and hematocrit levels.

A *P*-value of less than 0.05 was considered statistically significant. All statistical analysis was conducted using RStudio software (version 4.5.6, R Foundation for Statistical Computing, Vienna, Austria).

## 3 Results

### 3.1 Socio-demographic characteristics

A notable demographic observation is that the majority, or 64.5%, of patients were in the age group below 15 years. The median age calculated for this cohort is 12 years, with a wide range from the youngest at 2 years to the oldest at 43 years. In addition, most patients in this cohort were males (64.5%) (Supplementary Table S2).

### 3.2 LDH values among sickle cell patients during the three observation phases

During Baseline Phase of the study, a notable observation was the mean LDH level, which averaged at 649.73 U/L (±347.28). This measurement exceeded, the established normal LDH values (for women: 135–214 U/L, for men: 135–225 U/L, for children (2–15 years of age): 120–300 U/L, and for newborns (4–20 days of age): 225–600 U/L). Subsequently, in Phase 1, the average LDH values observed was 661.56 U/L (±367.39) also surpassing the value observed in Baseline Phase. In Phase 2, the mean LDH value was 529.90 U/L (±346.30). Although this value remained elevated relative to normal LDH levels - specifically, 216.7%, 220.3%, and 176.6% above baseline for baseline, Phase 1, and Phase 2, respectively - it represented a reduction compared to the levels observed in the previous phases (*p* = 0.001), as determined by the Wilcoxon test (Supplementary Table S3).

### 3.3 Distribution of LDH values in sickle-cell patients during the three observation phases

#### 3.3.1 Comparison of LDH between baseline phase and phase 1

Overall, LDH levels in Phase 1 were not significantly different from baseline (*p* = 0.349) as assessed by the Friedman test (Supplementary Table S4).

#### 3.3.2 LDH comparison between baseline and phase 2

In Phase 2, the LDH levels were significantly lower compared to baseline (*p* = 0.017) as assessed by the Friedman test (Supplementary Table S4).

#### 3.3.3 LDH comparison between phases 1 and 2

Also in phase 2, the LDH levels were significantly lower compared to baseline (*p* = 0.017) as assessed by the Friedman test (Supplementary Table S4).

### 3.4 Correlation between LDH and other blood count markers in sickle cell patients during the three observation phases

During the baseline assessment, by using the spearman test, there was a negative correlation observed between LDH values and parameters such as Hb (R = −0.304, −0.331 and −0.599) respectively for baseline, phase 1 and phase 2 and Hct (R = −0.283, −0.289 and −0.612) for baseline, phase 1 and phase 2, and a positive correlation with WBC (R = 0.274, 0.396 and 0.406) for baseline, phase 1 and phase 2. However, within the third phase, LDH exhibited a negative correlation solely with Hct (Supplementary Table S5). However, these associations were not statistically significant in phase 2 (Supplementary Table S5).

## 4 Discussions

This study was designed to evaluate the potential impact of L-arginine supplementation in conjunction with HU therapy on steady-state LDH levels in patients with SCD. Given the central role of LDH as a biomarker of hemolysis and endothelial dysfunction, the results of this study provide insight into the interplay between NO bioavailability and hemolytic markers, with potential implications for improving therapeutic strategies in the management of SCD. In the context of our study, which was designed to evaluate the potential impact of arginine supplementation on lactate dehydrogenase (LDH) levels in patients with stable sickle cell disease, several notable findings emerged.

LDH levels were persistently high throughout all three phases of the study, reinforcing its established role as a reliable hematologic marker of hemolysis in individuals with sickle cell disease (SCD). This finding is consistent with previous research, such as the work of Kato et al., who identified LDH as a robust biomarker reflecting hemolysis, hemoglobinemia, nitric oxide (NO) consumption and NO resistance in SCD patients (4). Notably, high serum LDH levels have been documented in SCD patients even during steady state, indicating the ongoing nature of hemolysis (12). The sustained elevation of LDH observed throughout our study highlights the chronic nature of hemolysis in SCD and underscores the critical need for therapeutic strategies targeting this persistent pathological process.

Second, a significant increase in LDH levels was observed after initiation of hydroxyurea (HU) treatment. This may be explained by the altered nitric oxide (NO) metabolism in sickle cell patients. In addition to its well-established role in increasing fetal hemoglobin (Hb F) levels, HU has been shown to increase NO bioavailability, thereby alleviating endothelial dysfunction and reducing vaso-occlusive events. These combined effects contribute to the overall reduction of sickle cell disease complications (7, 8). At the same time, LDH serves as a reliable biomarker not only for hemoglobinemia but also for NO consumption and resistance in SCD patients (13). The observed increase in LDH levels probably reflects the increased demand for NO bioavailability during HU treatment in these patients.

However, when arginine supplementation was added to hydroxyurea (HU) therapy, a significant reduction in LDH levels was observed. This suggests that arginine may serve as a valuable adjunctive treatment in the management of sickle cell disease (SCD). These findings are consistent with previous research, including studies by Morris et al., Lou et al., and Suboticki et al., which suggest that HU treatment may deplete arginine stores due to its enhancement of nitric oxide (NO) production (8, 20–23). The consistency of our results with the existing literature reinforces the observed reduction in LDH levels and highlights the complex interaction between HU therapy, arginine supplementation, and their potential therapeutic implications in the management of SCD.

Our findings also suggest that arginine supplementation may increase nitric oxide (NO) levels and potentially reduce vaso-occlusive episodes (8, 10). NO, a potent vasodilator, is critical in regulating vascular tone and maintaining endothelial function (4, 5). In sickle cell disease (SCD), there is a well-documented state of NO resistance and reduced NO bioavailability, primarily due to scavenging of NO by free hemoglobin released during hemolysis (4, 5). Since arginine is a major substrate for NO synthesis, it plays a critical role in counteracting NO resistance. The therapeutic potential of arginine supplementation in the management of SCD is further supported by previous studies demonstrating its efficacy in improving pain management and alleviating acute complications in SCD patients (8, 10).

Given the chronic and debilitating nature of sickle cell disease (SCD) and the limited availability of therapeutic options, particularly in resource-limited settings, arginine supplementation represents a promising adjunctive therapy. Our preliminary findings provide valuable insights into the potential benefits of arginine supplementation in the management of SCD, particularly its ability to reduce LDH levels and thereby mitigate hemolysis. These results highlight the potential for expanding research and clinical applications of arginine, particularly in regions where advanced treatment modalities are not readily available.

Interestingly, our study showed that high LDH levels were negatively correlated with key hematological markers, particularly hemoglobin (Hb) and hematocrit (Hct), while showing a positive correlation with WBC counts. This pattern was particularly evident at baseline and in the third phase of the study. The negative correlation between LDH and Hb is consistent with existing literature indicating that lower Hb levels are often associated with increased hemolysis, which naturally leads to elevated LDH levels (4, 12, 20, 21). Similarly, the inverse relationship between LDH and Hct is consistent with the observation that lower Hct levels are indicative of anemia, a condition often associated with higher LDH levels in SCD patients (4). The positive correlation between LDH and WBC is consistent with previous findings, such as those reported by Kato et al. (4). These correlations underscore the complex interactions between different hematological parameters in SCD. The intricate nature of these interactions highlights the need for a comprehensive, multidimensional approach to patient management. A deeper understanding of these correlations could provide critical insights into the pathophysiological mechanisms of the disease and inform the development of more targeted and effective therapeutic strategies.

It is important to emphasize that, to the best of our knowledge, this study is the first of its kind to be conducted in the Democratic Republic of Congo.

Several limitations must be considered when interpreting our preliminary results. First, the retrospective design introduces the potential for selection bias and the influence of unmeasured confounders. Second, it is important to recognize that our study does not establish a causal relationship between lactate dehydrogenase (LDH) levels and arginine supplementation, as the lack of a placebo group precludes definitive conclusions regarding the effects of arginine on LDH. Third, the generalizability of our findings is limited by the relatively small sample size and the focus on a single healthcare facility. However, it is worth noting that our sample size exceeds that of previous studies. For example, Morris et al. conducted a study in the United States with a cohort of only ten SCD patients to investigate the effect of arginine therapy on pulmonary arterial hypertension (PAH) (8, 9). Similarly, Darcielle et al. in Brazil evaluated L-arginine supplementation as an adjunct to hydroxyurea (HU) in the treatment of acute chest syndrome (ACS) in 21 adult patients, of whom only 12 received a combined regimen of HU and L-arginine (5). In addition, our study prioritized the analysis of biological markers over clinical manifestations, as we focused on patients in a steady state, unlike the studies by Kato, Morris, and others, which included patients during acute episodes or complications. To strengthen and validate these preliminary findings, future research should employ more robust study designs, such as randomized controlled trials with larger and more diverse sample sizes. In addition, evaluation of the long-term safety and efficacy of arginine supplementation is critical to its potential clinical application.

## 5 Conclusion

In summary, findings from this initial investigation provide insight into the potential benefits of using arginine supplementation as a component of SCD management, particularly in the context of reducing LDH levels. Although these findings are promising, their credibility and applicability require substantiation through additional and more extensive research efforts.

## Data Availability

The original contributions presented in the study are included in the article/Supplementary Material, further inquiries can be directed to the corresponding author.
