# Supplementary material for: Arginine supplementation improves lactate dehydrogenase levels in steady-state sickle cell patients: preliminary findings from Kinshasa, the Democratic Republic of Congo
Source: Front Pain Res (Lausanne). 2024 Nov 22;5:1391666. doi: 10.3389/fpain.2024.1391666 (PMC11621210; doi:10.3389/fpain.2024.1391666)
Supplement: Supplementary file 1 [file Table1.docx]

Supplementary Material

Article Title

Ange C M. Ngonde^1,2*^, Philippe N. Lukanu^1,2^, Ange Mubiala^3^, Michel N. Aloniuthor^4^

^1^Polyclinique de Kinshasa, Kinshasa, The Democratic Republic of Congo

^2^Department de Médecine de Famille et soins de santé primaires, Université Protestante du Congo, Kinshasa, The Democratic Republic of Congo

^3^Institut National de Recherche Biomédicale (INRB), Kinshasa, The Democratic Republic of Congo

^4^Département de Pédiatrie, Cliniques Universitaires de Kinshasa, Faculté de Médecine, Université de Kinshasa, Kinshasa , The Democratic Republic of Congo

*** Correspondence:** Ange Christian Mambakasa Ngonde* angondemc@gmail.com

# Supplementary Figures and Tables

For more information on Supplementary Material and for details on the different file types accepted, please see [here](https://www.frontiersin.org/guidelines/author-guidelines#supplementary-material).

## Supplementary Figures

**Table 1: Patient’s characteristics**

| **Patient’s characteristics** | **Total** | **Sex** | |
| --- | --- | --- | --- |
|  |  | **Female** | **Male** |
| **Age** |  |  |  |
| 0 - 14 years | 8,8 ± 3,56 | 6,6 ± 3,29 | 9,53 ± 3,46 |
|  | 20 (64,5) | 5 (45,5) | 15 (75,0) |
| 15+ years | 20,64 ± 8,38 | 18,0 ± 4,65 | 23,8 ± 11,21 |
|  | 11 (35,5) | 6 (54,5) | 5 (25,0) |
| Patient weight | 20,64 ± 8,38 | 18,0 ± 4,65 | 23,8 ± 11,21 |
| **Sex Ratio** | 1,81 |  |  |
| **Total** | 31 | 11 (35,5) | 20 (64,5) |
| **Patient Age** | | | |
| Mean age | 13,00 | Median | 12,00 |
| Standard deviation | 8,042 | Range | 41 |
| Minimum | 2 | Maximum | 43 |

**Table 2: LDH values among sickle cell patients during the three observation phases**

| **Patient’s characteristics** | **Total**  **n=31** | **Age** | | **P-value**  **(wilcoxon)** |
| --- | --- | --- | --- | --- |
|  |  | **0 – 14 years**  **n=20** | **15 years and upper**  **n=11** |  |
| **Sex** |  |  |  |  |
| Male | 13,10±8,68 | 9,53± 3,46 | 6,60±3,29 | 0,001 |
|  | 11,50 (41) | 11 (12) | 6 (7) |  |
| Female | 12,82±7,11 | 18,0±4,65 | 23,80±12,21 |  |
|  | 15,0 (24) | 16 (12) | 19 (28) |  |
| **LDH** |  |  |  |  |
| Baseline | 649,73 ± 347,28 | 632,81 ± 389,41 | 694,83 ± 219,70 | 0,001 |
| phase 1 | 661,56 ± 367,39 | 622,20 ± 240,50 | 720,60 ± 513,33 | 0,001 |
| phase 2 | 529,90 ± 346,3 | 500,13 ± 150,34 | 584,05 ± 558,40 | 0,001 |
| **Hb** |  |  |  |  |
| Baseline | 7,66±1,06 | 7,77±1,16 | 7,32±0,64 | 0,001 |
| Phase 1 | 7,96 ± 1,33 | 7,81±1,39 | 8,18±1,27 | 0,578 |
| Phase 2 | 7,71 ± 1,26 | 7,74±1,32 | 7,67±1,21 | 0,354 |
| **Hct** |  |  |  |  |
| Baseline | 22,86 ± 3,09 | 23,17±3,27 | 22,08 ±2,69 | 0,001 |
| Phase 1 | 23,74 ± 3,83 | 23,13±3,84 | 24,54±3,86 | 0,001 |
| Phase 2 | 23,01 ± 3,24 | 22,86±2,60 | 23,25±4,22 | 0,001 |
| **WBC** |  |  |  |  |
| Baseline | 12747,3± 3744 | 12762,5±3714,5 | 12706,7±4181 | 0,001 |
| Phase 1 | 10983,3 ± 4297,5 | 11914,3±4402,6 | 9680,0±3995,5 | 0,001 |
| Phase 2 | 11636,67 ± 3916 | 12515,8±4143,3 | 10118,2 ±3094,1 | 0,001 |

*LDH: Lactate dehydrogenase; Hb: Hemoglobin; Hct: Hematocrit; WBC: White blood cell*Normal LDH values: Female: 135 - 214 U/L; Male: 135 - 225 U/L; Children (2 to 15 years): 120 - 300 U/L; Newborns (4 to 20 days): 225 - 600 U/L.

*The difference between three phase are is significant.

**Table 3: Overall comparison of the 3 phases**

|  | ***P-value***  ***Friedman Test*** | ***Mean Difference*** | ***95 % confiance intervalleof the différence*** | |
| --- | --- | --- | --- | --- |
|  |  |  | ***lower*** | ***Upper*** |
| LDH_ baseline | 0,002* | 649,75 | 495,75 | 803,70 |
| LDH_TR1 |  | 661,56 | 509,9079 | 813,2121 |
| LDH_TR2 |  | 529,90 | 402,8788 | 656,9277 |

*P value <0.05.

**Table 4: Correlation between LDH and other biological markers (Hb, Hct and WBC) during 3 phases**

|  | Correlation Statistics | ***Hb*** | ***Hct*** | ***WBC*** |
| --- | --- | --- | --- | --- |
| ***LDH baseline*** |  |  |  |  |
|  | ***Spearman Rho*** | -0,304^*^ | -0,283^*^ | 0,274* |
|  | ***P-value*** | 0,008 | 0,015 | 0,017 |
| ***LDH_Traitement1*** |  |  |  |  |
|  | ***Spearman Rho*** | -0,331^ns^ | -0,289 ^ns^ | 0,396 ^ns^ |
|  | ***P-value*** | 0,142 | 0,203 | 0,068 |
| ***LDH_Traitement2*** |  |  |  |  |
|  | ***Spearman Rho*** | -0,599* | -0,612* | 0,406* |
|  | ***P-value*** | ,153 | ,006 | 0,903 |

*P<0.05

**
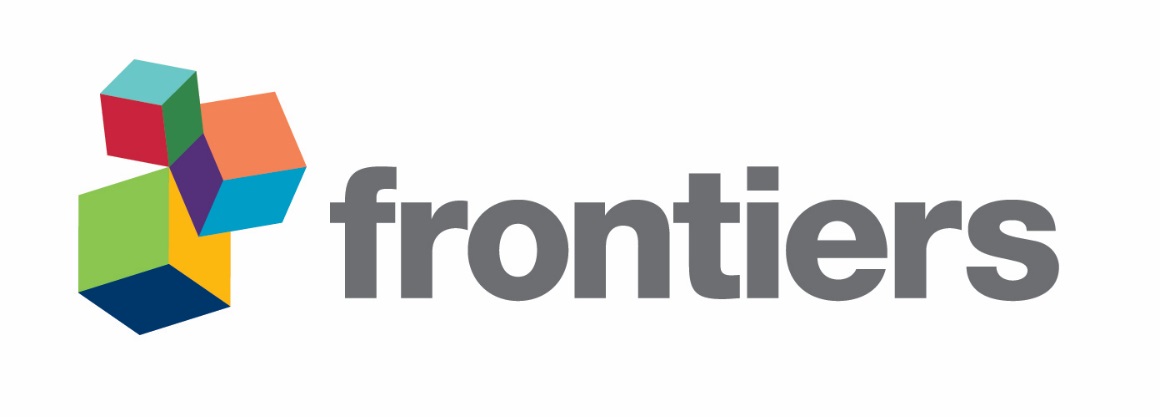
**

**Supplementary Figure 1.** The figure legends are required to have the same font as the main text, 12 point normal Times New Roman, single spaced. Please use a single paragraph for each legend and prepare the figures keeping in mind the PDF layout.
