# Supplementary material for: Arginine supplementation improves lactate dehydrogenase levels in steady-state sickle cell patients: preliminary findings from Kinshasa, the Democratic Republic of Congo
Source: Front Pain Res (Lausanne). 2024 Nov 22;5:1391666. doi: 10.3389/fpain.2024.1391666 (PMC11621210; doi:10.3389/fpain.2024.1391666)
Supplement: Supplementary file 3 [file Table3.docx]

Supplementary Material

Article Title

Ange C M. Ngonde^1,2*^, Philippe N. Lukanu^1,2^, Ange Mubiala^3^, Michel N. Aloniuthor^4^

^1^Polyclinique de Kinshasa, Kinshasa, The Democratic Republic of Congo

^2^Department de Médecine de Famille et soins de santé primaires, Université Protestante du Congo, Kinshasa, The Democratic Republic of Congo

^3^Institut National de Recherche Biomédicale (INRB), Kinshasa, The Democratic Republic of Congo

^4^Département de Pédiatrie, Cliniques Universitaires de Kinshasa, Faculté de Médecine, Université de Kinshasa, Kinshasa, The Democratic Republic of Congo

*** Correspondence:** Ange Christian Mambakasa Ngonde* angondemc@gmail.com

# Supplementary Figures and Tables

For more information on Supplementary Material and for details on the different file types accepted, please see [here](https://www.frontiersin.org/guidelines/author-guidelines#supplementary-material).

## Supplementary Figures

**Table 1: Patient’s characteristics**

| **Patient’s characteristics** | **Total**  **N=31 (100%)** | **Sex** | |
| --- | --- | --- | --- |
|  |  | **Female**  **n=11 (35,5% )** | **Male**  **n=20 (64,5%)** |
| **Age** |  |  |  |
| Mean ± Sd | 13,00 ± 8,042 |  |  |
| Median and quartiles | 12,00 (9,00 – 16,00) |  |  |
| Age group |  |  |  |
| 0 - 14 years | 8,8 ± 3,56 | 6,6 ± 3,29 | 9,53 ± 3,46 |
|  | 20 (64,5) | 5 (45,5) | 15 (75,0) |
| 15+ years | 20,64 ± 8,38 | 18,0 ± 4,65 | 23,8 ± 11,21 |
|  | 11 (35,5) | 6 (54,5) | 5 (25,0) |
| Patient weight | 20,64 ± 8,38 | 18,0 ± 4,65 | 23,8 ± 11,21 |
| **Sex Ratio** | 1,81 |  |  |

**Table 2: Overall comparison of biological parameters and LDH in three phases**

|  | Base line  (LDH) | Phase 1  (LDH+HU) | Phase 2  (LDH, HU and ARG) | P-value |
| --- | --- | --- | --- | --- |
| Hb | 7,71 ± 1,09 | 8,23 ± 1,17 | 8,13 ± 0,85 | 0,050 |
| Hct | 23,28 ± 2,74 | 24,63 ± 2,89 | 23,83 ± 2,67 | 0,053 |
| Gb | 12517,14 ± 4272,78 | 10528,57 ± 4042,16 | 10871,43 ± 3481,03 | 0,009 |
| LDH | 649,73 ± 347,28 | 661,56 ± 367,39 | 529,87 ± 346,31 | 0,002 |
| % LDHnorm  (120 - 300 U/L) | 116,7 | 120,5 | 76 |  |
| p-value : test de Friedman | | | | |

**Table 3: pairwise comparison of biological parameters and LDH**

| Parameters | Phases | p-value |
| --- | --- | --- |
| LDH | Base line (LDH) | 0,617 |
|  | Phase 1 (LDH+HU) |  |
|  | Base line (LDH) | 0,040 |
|  | Phase 2 (LDH, HU and ARG) |  |
|  | Phase 1 (LDH+HU) | 0,011 |
|  | Phase 2 (LDH, HU and ARG) |  |
| Hb | Base line (LDH) | 0,328 |
|  | Phase 1 (LDH+HU) |  |
|  | Base line (LDH) | 0,954 |
|  | Phase 2 (LDH, HU and ARG) |  |
|  | Phase 1 (LDH+HU) | 0,329 |
|  | Phase 2 (LDH, HU and ARG) |  |
| Hct | Base line (LDH) | 0,245 |
|  | Phase 1 (LDH+HU) |  |
|  | Base line (LDH) | 0,300 |
|  | Phase 2 (LDH, HU and ARG) |  |
|  | Phase 1 (LDH+HU) | 0,753 |
|  | Phase 2 (LDH, HU and ARG) |  |
| Gb | Base line (LDH) | 0,003 |
|  | Phase 1 (LDH+HU) |  |
|  | Base line (LDH) | 0,090 |
|  | Phase 2 (LDH, HU and ARG) |  |
|  | Phase 1 (LDH+HU) | 0,465 |
|  | Phase 2 (LDH, HU and ARG) |  |
| P-value : test de Will Coxon | | |

*LDH: Lactate dehydrogenase; Hb: Hemoglobin; Hct: Hematocrit; WBC: White blood cell*Normal LDH values: Female: 135 - 214 U/L; Male: 135 - 225 U/L; Children (2 to 15 years): 120 - 300 U/L; Newborns (4 to 20 days): 225 - 600 U/L.

*The difference between three phase are is significant.

**Table 4: Correlation between LDH and other biological markers (Hb, Hct and WBC) during 3 phases**

|  | Correlation Statistics | ***Hb*** | ***Hct*** | ***WBC*** |
| --- | --- | --- | --- | --- |
| ***LDH baseline*** |  |  |  |  |
|  | ***Spearman Rho*** | -0,246 | -0,232 | 0,396 |
|  | ***P-value*** | 0,123 | 0,152 | 0,068 |
| ***LDH_***(LDH+HU) |  |  |  |  |
|  | ***Spearman Rho*** | -0,599^*^ | -0,612^*^ | 0,406 |
|  | ***P-value*** | 0,002 | 0,002 | 0,048^*^ |
| ***LDH*** (LDH, HU and ARG) |  |  |  |  |
|  | ***Spearman Rho*** | -0,105 | -0,098 | 0,083 |
|  | ***P-value*** | 0,579 | 0,625 | 0,580 |

*P<0.05

**Supplementary Figure 1.** The figure legends are required to have the same font as the main text, 12 point normal Times New Roman, single spaced. Please use a single paragraph for each legend and prepare the figures keeping in mind the PDF layout.
