# Supplementary material for: Arginine supplementation improves lactate dehydrogenase levels in steady-state sickle cell patients: preliminary findings from Kinshasa, the Democratic Republic of Congo
Source: Front Pain Res (Lausanne). 2024 Nov 22;5:1391666. doi: 10.3389/fpain.2024.1391666 (PMC11621210; doi:10.3389/fpain.2024.1391666)
Supplement: Supplementary file 4 [file Table4.docx]

Supplementary Material

Article Title

Ange C M. Ngonde^1,2*^, Philippe N. Lukanu^1,2^, Ange Mubiala^3^, Michel N. Aloniuthor^4^

^1^Polyclinique de Kinshasa, Kinshasa, The Democratic Republic of Congo

^2^Department de Médecine de Famille et soins de santé primaires, Université Protestante du Congo, Kinshasa, The Democratic Republic of Congo

^3^Institut National de Recherche Biomédicale (INRB), Kinshasa, The Democratic Republic of Congo

^4^Département de Pédiatrie, Cliniques Universitaires de Kinshasa, Faculté de Médecine, Université de Kinshasa, Kinshasa , The Democratic Republic of Congo

*** Correspondence:** Ange Christian MambakasaNgonde* angondemc@gmail.com

# Supplementary Figures and Tables

For more information on Supplementary Material and for details on the different file types accepted, please see [here](https://www.frontiersin.org/guidelines/author-guidelines#supplementary-material).

## Supplementary Figures

**Table 1: Inclusion and exclusion criteria**

| Inclusion criteria | Exclusion criteria |
| --- | --- |
| LDH assay data obtained from individuals diagnosed with SCD:  -These individuals were followed on an outpatient basis during the designated study period.  -The selection process was contingent upon several specific requirements, including the availability of LDH assay measurements obtained outside of crisis episodes.  -The presence of at least two LDH assay values documented during two distinct follow-up periods. These periods were divided into two primary categories: those occurring optionally before the initiation of HU treatment and those during HU treatment. An additional mandatory requirement was that LDH assay measurements be available during the concurrent administration of HU and arginine-containing supplement (ARG). | - LDH data collected during crisis episodes were excluded.  - In addition, records without dosing measurements during the third follow-up period were considered ineligible for inclusion.  - In addition, cases with dosage measurements limited to only one follow-up period were excluded from the study analysis. . |

**Table 2: Patient’s characteristics**

| **Patient’s characteristics** | **Total** | **Patient Age group** | |
| --- | --- | --- | --- |
|  |  | 0 - 14 years | 15+ years |
| **Sex** |  |  |  |
| **Female** | 20.64 ± 8.38 | 6.6 ± 3.29 | 18.0 ± 4.65 |
|  | 11 (35.5) | 5 (45.5) | 6 (54.5) |
| **Male** | 8.8 ± 3.56 | 9.53 ± 3.46 | 23.8 ± 11.21 |
|  | 20 (64.5) | 15 (75.0) | 5 (25.0) |
| **Patient weight** | 20.64 ± 8.38 | 18.6 ± 6.2 | 40.4 ± 9.7 |
| **LDH** Baseline | 649.73 ± 347.28 | 632.81 ± 389.41 | 694.83 ± 219.70 |
| **Total** | 31 | 11 (35.5) | 20 (64.5) |
| **Patient Age** | | | |
| Mean age& SD | 13.00± 8.04 |  |  |
| Median& quatiles | 12.00 (9.00 – 16.00) |  |  |
| Minimum | 2 |  |  |
| Maximum | 43 |  |  |

**Table 3: LDH values among sickle cell patients during the three observation phases**

| **Patient’s characteristics** | **Total**  **n=31** | **Age** | | **P-value**  **Wilcoxon test** |
| --- | --- | --- | --- | --- |
|  |  | **0 – 14 years**  **n=20** | **15 years and upper**  **n=11** |  |
| **Sex** |  |  |  |  |
| Male | 13.10±8.68 | 9.53± 3.46 | 6.60±3.29 | 0.001 |
|  | 11.50 (41) | 11 (12) | 6 (7) |  |
| Female | 12.82±7,11 | 18,0±4,65 | 23,80±12,21 |  |
|  | 15.0 (24) | 16 (12) | 19 (28) |  |
| **LDH** |  |  |  |  |
| Baseline | 649.73 ± 347.28 | 632.81 ± 389.41 | 694.83 ± 219.70 | 0.001 |
| %LDHnle | 216.7%, |  |  |  |
| phase 1 | 661.56 ± 367.39 | 622.20 ± 240.50 | 720.60 ± 513.33 | 0.001 |
| %LDHnle | 220.3%, |  |  |  |
| phase 2  %LDHnle | 529.90 ± 346.3  176.6% | 500.13 ± 150.34 | 584.05 ± 558.40 | 0.001 |
| **Hb** |  |  |  |  |
| Baseline | 7.66±1.06 | 7.77±1.16 | 7.32±0.64 | 0.001 |
| Phase 1 | 7.96 ± 1.33 | 7.81±1.39 | 8.18±1.27 | 0.578 |
| Phase 2 | 7.71 ± 1.26 | 7.74±1.32 | 7.67±1.21 | 0.354 |
| **Hct** |  |  |  |  |
| Baseline | 22.86 ± 3.09 | 23.17±3.27 | 22.08 ±2.69 | 0.001 |
| Phase 1 | 23.74 ± 3.83 | 23.13±3.84 | 24.54±3.86 | 0.001 |
| Phase 2 | 23.01 ± 3.24 | 22.86±2.60 | 23.25±4.22 | 0.001 |
| **WBC** |  |  |  |  |
| Baseline | 12747.3± 3744 | 12762.5±3714.5 | 12706.7±4181 | 0.001 |
| Phase 1 | 10983.3 ± 4297,5 | 11914.3±4402.6 | 9680.0±3995.5 | 0.001 |
| Phase 2 | 11636.6 ± 3916 | 12515.8±4143.3 | 10118.2 ±3094.1 | 0.001 |

*LDH: Lactate dehydrogenase; Hb: Hemoglobin; Hct: Hematocrit; WBC: White blood cell*Normal LDH values: Female: 135 - 214 U/L; Male: 135 - 225 U/L; Children (2 to 15 years): 120 - 300 U/L; Newborns (4 to 20 days): 225 - 600 U/L.

*The difference between three phase is significant (p-value mann Whitney).

**Table 4: LDH comparison in all 3 phases**

|  | *P-value* | *Mean Difference* | *95 % confiance intervalle of the différence* | |
| --- | --- | --- | --- | --- |
|  |  |  | *lower* | *Upper* |
| LDH_ baseline | 0.002** | 649.75 | 495.75 | 803.70 |
| LDH Phase 1 |  | 661.56 | 509.9079 | 813.2121 |
| LDH Phase 2 |  | 529.90 | 402.8788 | 656.9277 |
| LDH Baseline | 0.349 | 347,284 | 234.00 | 1907.00 |
| LDH Phase 1 |  | 367,39228 | 193.00 | 1715.00 |
| LDH Baseline | 0.017* | 649.727 | 495.75 | 803.70 |
| LDH Phase 2 |  | 529.90323 | 402.8788 | 656.9277 |
| LDHPhase 1 | 0.017* | 649.727 | 495.75 | 803.70 |
| LDH Phase 2 |  | 529.90323 | 402.8788 | 656.9277 |

**P value(*Friedman Test*)<0.05. With P=0,002, the difference in LDH in all 3 phases is statistically significant

*P value(*Wilcoxon Test*)<0.05, the difference in LDH between these 2 phases is statistically significant

**Table 5: Correlation between LDH and other biological markers (Hb, Hct and WBC) during 3 phases**

|  | Correlation Statistics | ***Hb*** | ***Hct*** | ***WBC*** |
| --- | --- | --- | --- | --- |
| ***LDH baseline*** |  |  |  |  |
|  | ***Spearman Rho*** | -0.304^*^ | -0.283^*^ | 0.274* |
|  | ***P-value*** | 0.008 | 0.015 | 0.017 |
| ***LDH_ Phase1*** |  |  |  |  |
|  | ***Spearman Rho*** | -0.331^ns^ | -0.289 ^ns^ | 0.396 ^ns^ |
|  | ***P-value*** | 0.142 | 0.203 | 0.068 |
| ***LDH_ Phase2*** |  |  |  |  |
|  | ***Spearman Rho*** | -0,599* | -0,612* | 0.406* |
|  | ***P-value*** | ,153 | ,006 | 0.903 |

*P<0.05

**
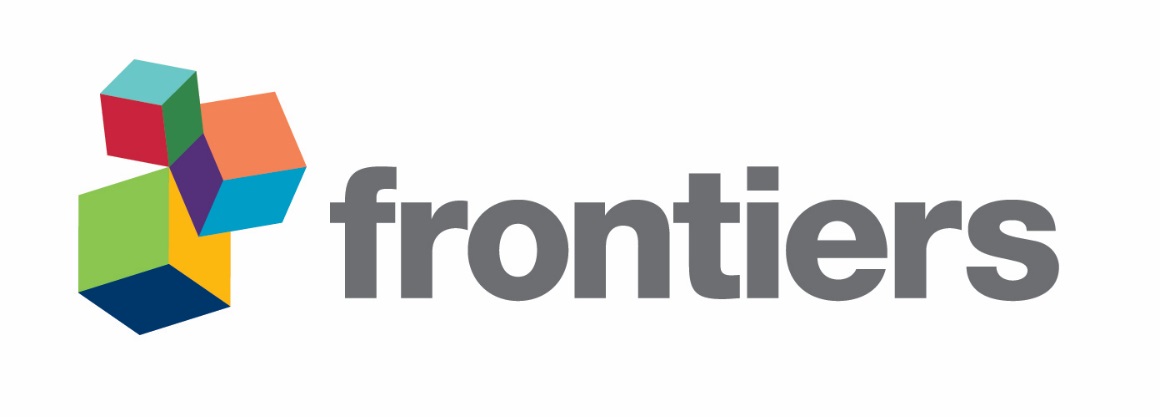
**

**Supplementary Figure 1.** The figure legends are required to have the same font as the main text, 12 point normal Times New Roman, single spaced. Please use a single paragraph for each legend and prepare the figures keeping in mind the PDF layout.
