# Supplementary material for: Arginine supplementation improves lactate dehydrogenase levels in steady-state sickle cell patients: preliminary findings from Kinshasa, the Democratic Republic of Congo
Source: Front Pain Res (Lausanne). 2024 Nov 22;5:1391666. doi: 10.3389/fpain.2024.1391666 (PMC11621210; doi:10.3389/fpain.2024.1391666)
Supplement: Supplementary file 6 [file Table6.docx]

Supplementary Material

Article Title

Ange C M. Ngonde^1,2*^, Philippe N. Lukanu^1,2^, Ange Mubiala^3^, Michel N. Aloniuthor^4^

^1^Polyclinique de Kinshasa, Kinshasa, The Democratic Republic of Congo

^2^Department de Médecine de Famille et soins de santé primaires, Université Protestante du Congo, Kinshasa, The Democratic Republic of Congo

^3^Institut National de Recherche Biomédicale (INRB), Kinshasa, The Democratic Republic of Congo

^4^Département de Pédiatrie, Cliniques Universitaires de Kinshasa, Faculté de Médecine, Université de Kinshasa, Kinshasa , The Democratic Republic of Congo

*** Correspondence:** Ange Christian MambakasaNgonde* angondemc@gmail.com

# Supplementary Figures and Tables

For more information on Supplementary Material and for details on the different file types accepted, please see [here](https://www.frontiersin.org/guidelines/author-guidelines#supplementary-material).

## Supplementary Figures

**Table 1: Inclusion and exclusion criteria**

| Inclusion criteria | Exclusion criteria |
| --- | --- |
| LDH assay data were collected from individuals, aged at least 2 years and diagnosed with major sickle cell syndrome (SCD) (SS, SC or Sbthal) who were followed as outpatients during the designated study period.  - The selection process adhered to specific inclusion criteria, including the availability of LDH assay measurements taken outside of crisis episodes.  - In addition, participants were required to have at least two documented LDH assay values obtained during two different follow-up periods: either before initiation of hydroxyurea (HU) treatment or during HU treatment.  - Another mandatory criterion was the availability of LDH assay measurements during concomitant administration of HU and arginine-containing supplement (ARG). | - LDH data collected during crisis episodes were excluded.  - In addition, records without dosing measurements during the third follow-up period were considered ineligible for inclusion.  - In addition, cases with dosing measurements available for only a single follow-up period were excluded from the study analysis. |

**Table 2: Patient’s characteristics**

| **Patient’s characteristics** | **Total** | **Patient Age group** | |
| --- | --- | --- | --- |
|  |  | 0 - 14 years | 15+ years |
| **Sex** |  |  |  |
| **Female** | 20.64 ± 8.38 | 6.6 ± 3.29 | 18.0 ± 4.65 |
|  | 11 (35.5) | 5 (45.5) | 6 (54.5) |
| **Male** | 8.8 ± 3.56 | 9.53 ± 3.46 | 23.8 ± 11.21 |
|  | 20 (64.5) | 15 (75.0) | 5 (25.0) |
| **Patient weight** | 20.64 ± 8.38 | 18.6 ± 6.2 | 40.4 ± 9.7 |
| **LDH** Baseline | 649.73 ± 347.28 | 632.81 ± 389.41 | 694.83 ± 219.70 |
| **Total** | 31 | 11 (35.5) | 20 (64.5) |
| **Patient Age** | | | |
| Mean age& SD | 13.00± 8.04 |  |  |
| Median& quatiles | 12.00 (9.00 – 16.00) |  |  |
| Minimum | 2 |  |  |
| Maximum | 43 |  |  |

**Table 3: LDH values among sickle cell patients during the three observation phases**

| **Patient’s characteristics** | **Total**  **n=31** | **Age** | | **P-value**  **Wilcoxon test** |
| --- | --- | --- | --- | --- |
|  |  | **0 – 14 years**  **n=20** | **15 years and upper**  **n=11** |  |
| **Sex** |  |  |  |  |
| Male | 13.10±8.68 | 9.53± 3.46 | 6.60±3.29 | 0.001 |
|  | 11.50 (41) | 11 (12) | 6 (7) |  |
| Female | 12.82±7,11 | 18,0±4,65 | 23,80±12,21 |  |
|  | 15.0 (24) | 16 (12) | 19 (28) |  |
| **LDH** |  |  |  |  |
| Baseline | 649.73 ± 347.28 | 632.81 ± 389.41 | 694.83 ± 219.70 | 0.001 |
| %LDHnle | 216.7%, |  |  |  |
| phase 1 | 661.56 ± 367.39 | 622.20 ± 240.50 | 720.60 ± 513.33 | 0.001 |
| %LDHnle | 220.3%, |  |  |  |
| phase 2  %LDHnle | 529.90 ± 346.3  176.6% | 500.13 ± 150.34 | 584.05 ± 558.40 | 0.001 |
| **Hb** |  |  |  |  |
| Baseline | 7.66±1.06 | 7.77±1.16 | 7.32±0.64 | 0.001 |
| Phase 1 | 7.96 ± 1.33 | 7.81±1.39 | 8.18±1.27 | 0.578 |
| Phase 2 | 7.71 ± 1.26 | 7.74±1.32 | 7.67±1.21 | 0.354 |
| **Hct** |  |  |  |  |
| Baseline | 22.86 ± 3.09 | 23.17±3.27 | 22.08 ±2.69 | 0.001 |
| Phase 1 | 23.74 ± 3.83 | 23.13±3.84 | 24.54±3.86 | 0.001 |
| Phase 2 | 23.01 ± 3.24 | 22.86±2.60 | 23.25±4.22 | 0.001 |
| **WBC** |  |  |  |  |
| Baseline | 12747.3± 3744 | 12762.5±3714.5 | 12706.7±4181 | 0.001 |
| Phase 1 | 10983.3 ± 4297.5 | 11914.3±4402.6 | 9680.0±3995.5 | 0.001 |
| Phase 2 | 11636.6 ± 3916 | 12515.8±4143.3 | 10118.2 ±3094.1 | 0.001 |

*LDH: Lactate dehydrogenase; Hb: Hemoglobin; Hct: Hematocrit; WBC: White blood cell*Normal LDH values: Female: 135 - 214 U/L; Male: 135 - 225 U/L; Children (2 to 15 years): 120 - 300 U/L; Newborns (4 to 20 days): 225 - 600 U/L.

*The difference between three phase is significant (p-value mann Whitney).

**Table 4: LDH comparison in all 3 phases**

|  | *P-value* | *Mean Difference* | *95 % confiance intervalle of the différence* | |
| --- | --- | --- | --- | --- |
|  |  |  | *lower* | *Upper* |
| LDH_ baseline | 0.002** | 649.75 | 495.75 | 803.70 |
| LDH Phase 1 |  | 661.56 | 509.9079 | 813.2121 |
| LDH Phase 2 |  | 529.90 | 402.8788 | 656.9277 |
| LDH Baseline | 0.349 | 347,284 | 234.00 | 1907.00 |
| LDH Phase 1 |  | 367,39228 | 193.00 | 1715.00 |
| LDH Baseline | 0.017* | 649.727 | 495.75 | 803.70 |
| LDH Phase 2 |  | 529.90323 | 402.8788 | 656.9277 |
| LDH Phase 1 | 0.017* | 649.727 | 495.75 | 803.70 |
| LDH Phase 2 |  | 529.90323 | 402.8788 | 656.9277 |

**P value(*Friedman Test*)<0.05. With P=0,002, the difference in LDH in all 3 phases is statistically significant

*P value(*Wilcoxon Test*)<0.05, the difference in LDH between these 2 phases is statistically significant

**Table 5: Correlation between LDH and other biological markers (Hb, Hct and WBC) during 3 phases**

|  | Correlation Statistics | ***Hb*** | ***Hct*** | ***WBC*** |
| --- | --- | --- | --- | --- |
| ***LDH baseline*** |  |  |  |  |
|  | ***Spearman Rho*** | -0.304^*^ | -0.283^*^ | 0.274* |
|  | ***P-value*** | 0.008 | 0.015 | 0.017 |
| ***LDH_ Phase1*** |  |  |  |  |
|  | ***Spearman Rho*** | -0.331^ns^ | -0.289 ^ns^ | 0.396 ^ns^ |
|  | ***P-value*** | 0.142 | 0.203 | 0.068 |
| ***LDH_ Phase2*** |  |  |  |  |
|  | ***Spearman Rho*** | -0.599* | -0.612* | 0.406* |
|  | ***P-value*** | .153 | .006 | 0.903 |

*P<0.05

**
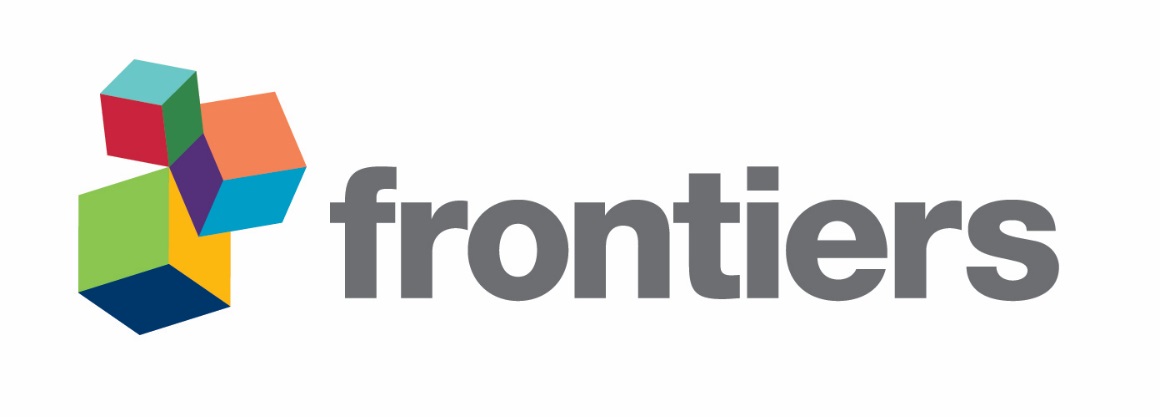
**

**Supplementary Figure 1.** The figure legends are required to have the same font as the main text, 12 point normal Times New Roman, single spaced. Please use a single paragraph for each legend and prepare the figures keeping in mind the PDF layout.
