# Supplementary material for: Arginine supplementation improves lactate dehydrogenase levels in steady-state sickle cell patients: preliminary findings from Kinshasa, the Democratic Republic of Congo
Source: Front Pain Res (Lausanne). 2024 Nov 22;5:1391666. doi: 10.3389/fpain.2024.1391666 (PMC11621210; doi:10.3389/fpain.2024.1391666)
Supplement: Supplementary file 8 [file Table8.docx]

**Schema of international longitudinal study of Arginine supplementation and its effect on improving LDH levels in steady-state sickle cell disease (SCD)patients**


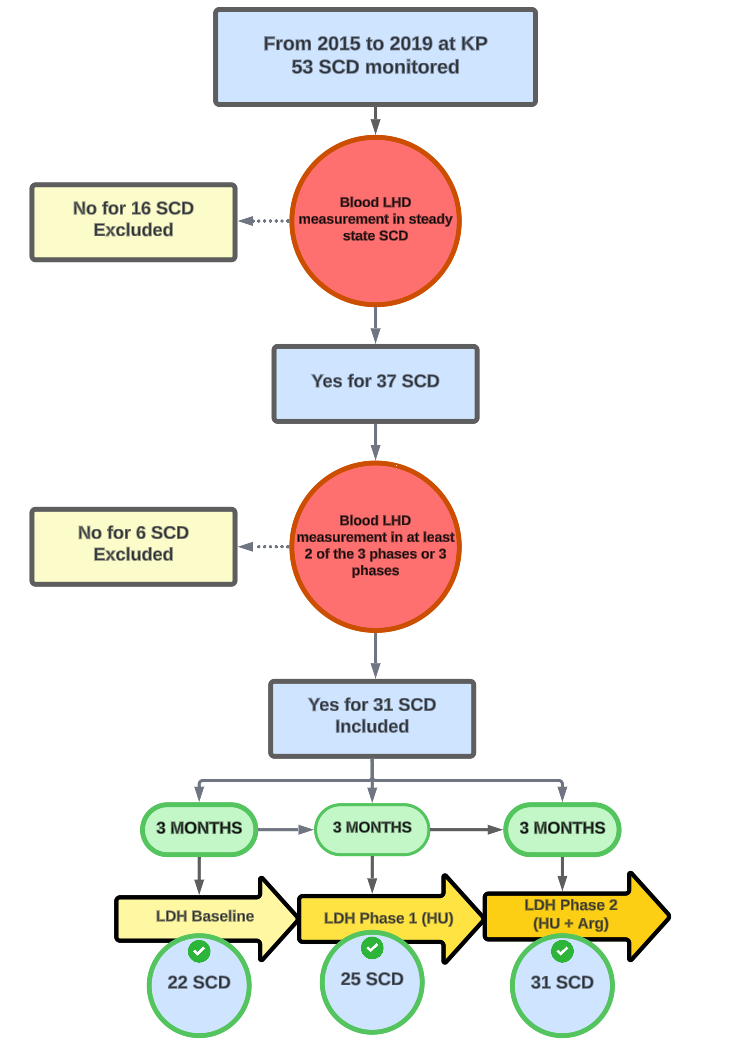


Follow up

LDH Data collected during crisis episode

Without dosing LDH measurement during third follow up period OR available for only a single follow up period
