# Supplementary material for: Arginine supplementation improves lactate dehydrogenase levels in steady-state sickle cell patients: preliminary findings from Kinshasa, the Democratic Republic of Congo
Source: Front Pain Res (Lausanne). 2024 Nov 22;5:1391666. doi: 10.3389/fpain.2024.1391666 (PMC11621210; doi:10.3389/fpain.2024.1391666)
Supplement: Supplementary file 9 [file Datasheet1.pdf]

Answers to Reviewers

	Evaluation	Answer
Reviewer 1	1.The authors state that there was a significant difference in the levels of LDH between Phase 1 to Phase 2 and Phase 2 to phase 3. The P-values, however, are approximately 0.5 for these comparisons which is not consistent with statistical significance. Therefore the interpretation of this data appears to be incorrect.<br><br>2.The introduction and discussion sections are too long and should be made more concise.<br><br>3.There is no power calculation or sample size justification presented so it is not clear if the lack of statistical significance is simply due to an inadequate number of study participants.<br><br>4. The manuscript would benefit from English language editing.	1.We thank you for your comments and have changed the statistical test. See line 143 to 147. So the Friedman test was used to perform a comparative analysis of LDH levels within the context of 3 groups observed at three different time points. The Spearman rank correlation test was used to assess the degree of association between LDH levels and hemoglobin levels, as well as between LDH and hematocrit levels. Pairwise comparisons of LDH levels were performed using the Wilcoxon signed-rank test. And A P-value of less than 0.05 was considered statistically significant.<br><br>2. We have taken this comment into account and in the new version; we have reduced the introduction (line 30 to 91) and made the discussion much more concise (line 183 to 265).<br><br>3. We carried out a retrospective study on the cohort of patients followed in this health facility during the study period.<br><br>4. In the new version, the entire manuscript has been revised in English.
Reviewer 2	Major comment:<br>– The sample size is small, and there is no placebo group to compare the effect of arginine supplementation to.	- This is the first observation in the cohort of patients followed in this health establishment during the study period. At least the comparison was made within the same group by monitoring the LDH markers in 3 successive periods, namely before taking the background medication (HU), during the intake of this background medication (HU) and during taking the basic treatment accompanied by the supplement (Arginine). The next studies will consist of comparing with control groups.

	– In general, the manuscript is not well written, and the authors are not up to date on treatments available for managing SCD. They claimed in line 58 that hydroxyurea is the only oral therapy for SCD. This is not correct, as there are two other oral disease-modifying medications for SCD (Oxbryta/Voxelotor, approved by the FDA in 2019, and Endari/L-glutamine oral powder, approved in 2017).<br><br>– The supplementary file only has Tables 1-4, while the authors referred to Tables 9–11, which were not part of the supplementary material or the main manuscript.<br><br>– Also, certain parts of the results were written in French. The citations did not follow any referencing styles or conventions, consisting of both English and French.	-Please note the improvements in the new version submitted. The statement in line 58 (old version) that "HU is the only oral treatment" has been replaced in line 56 (new version) by "one of the well-established interventions in the treatment of sickle cell disease... ". At the current stage of sickle cell disease management in Africa, we recognize that -HU is the only oral therapy for SCD in sub-Saharan Africa or better in low-income countries. We also add L-Glutamine and Voxelotor approved respectively in 2017 and 2019 by FDA available in the USA and under study in certain EU countries.<br><br>-This error has been corrected in the new version because it only has 4 tables in the supplementary materials.<br><br>-This error has been corrected in the new version because all the work is written in Englishcfr nouvelle version
Reviewer 3	Report should be improved with the following clarifications:<br>1) Line 42: Aim, needs to be rephrased clarified and add a sentence for rationale.<br><br>2) In 4.3 line 176: “While the significant difference between baseline LDH and treatment LDH supports the latter's increase, the latter contributes to the decrease…”<br>This Sentence is confusing, what is the author refering to as”treatment” HU? HU with arginine?<br><br>3) Conclusion line 301: ….the LDH value increases significantly, particularly in patients over 15 years of age	1)The Aim has been reworded and reduced from 9 lines to 5 lines. The study is justified by the sentence which shows the benefit of research on Arginine supplementation in a low-income country with a high prevalence of Sickle Cell Disease.<br><br>2) In the new version point 4.3 is replaced by 3.3. It is a question of observing the variation of LDH during the three observation phases. Thus using the Fredman Test, the LDH value is significantly different in the 3 phases (p= 0.002).<br><br>3) In the new version, we have removed this observation taking into account certain factors limiting our study. Particularly the small size

	and in male patients, due to the fact that HU induces NO production and may well deplete Arg reserves.<br><br>Can authors expand on this statement and explain why this statement is important in this study?	of our samples and the fact of only considering patients from a single health establishment.
Reviewer 4	-However, what is changes in LDH mean in terms of clinical presentation as well as treatment outcomes is not clear.<br>-Given the enormous economic and health burden of sickle-cell anemia, especially on developing countries with limited resources, the finding can be of interest for future studies to develop alternative and affordable intervention modality for sickle-cell anemia. Considering the role of malnutrition in increased risk of sickle-cell disease, for example, low arginine and nitric oxide bioavailability are implicated in morbidity related to sickle-cell disease, simple interventions are sensible, especially in low-income settings. This study hits that arginine supplementation may benefit patients with chronic hemolysis and improve, as an adjunct therapy, the action of current therapeutics. However, there are major concerns in the study design that limit making such conclusions. Therefore, I recommend rejection.<br><br>Major concern:<br>1. The three different periods of medical follow are not consistent in duration where the patients were treated for at least three months. In other words, the patients may have minimum of three months of treatment, but the duration may vary within and between groups. This can be a source of variability as the duration of treatment is not constant.<br><br>2. The study has two age groups (1-14 and >15 years of age). However, the rationale for such classification was not	- Observations within the framework of this study focused on the biological variation of LDH during the 3 phases of treatment; on the other hand, the clinical effects of the reduction in LDH could not be exploited and could be exploited within the framework of a future prospective study.<br><br>Major concern<br>1° It should be noted that the duration of monitoring in each phase is 3 months, i.e. we retained the LDH values, in stable state, for 3 months for each patient. In this sense, the observation time in a given phase is the same for each patient.<br><br>2°The classification into two age groups (1-14 years and >15 years) is dependent on the LDH dosage, the variation of which follows

	well established.	these 2 age groups. Cfr the manufacturer or the calibration of the test gives the normal values according to this age category.<br>Quantification of LDH levels was performed using the Cobas c 111 automated analyzer. This automated system adheres to established reference ranges, standardized at a temperature of 37°C as follows: for women: 135 - 214 U/L,for men: 135 - 225 U/L,for children (2 to 15 years of age): 120 - 300 U/L, and for newborns (4 to 20 days of age): 225 - 600 U/L.
	3. The cut-off age for arginine dosing is not well justified (i.e., why is the first group (1-14 years of age) receives 500 mg and the second group (for >15 years) receives 1000 mg. The latter received double dose of the former. This is another source for variation and can confound the results.	3° On the one hand the dosage of HU is a function of the patient's weight and on the other hand the metabolism of HU requires consumption of NO substrate (Arg). Since those over 15 years old need a high dose of HU based on their weight, this is why the dose of Arg is doubled for this age group.
	4. Lastly, the paper is poorly written. It is repetitive and lacks cohesive flow.	4° We have greatly improved the writing of the new version of the manuscript.
	Minor concerns:<br>1. Small sample size.	Minor:<br>1. The sample size is small: certainly constitutes a limitation of our study but it has the merit of being among the first observations in our environment and appears superior to other studies in comparison on Arginine in the Sickle cell disease as referenced in the discussion and conclusion of this new version of the manuscript;
	2. The reference list is only 18 papers suggesting the review of literature may not be through.	2. We have extended to 20 articles but it is necessary to recognize the weakness of the literature in relation to the use of Arginine under LDH monitoring in Sickle Cell Disease.
	3. There are also several typos and grammatical errors too numerous to list.	3. We have greatly improved the writing of the new version of the manuscript
