# Supplementary material for: Arginine supplementation improves lactate dehydrogenase levels in steady-state sickle cell patients: preliminary findings from Kinshasa, the Democratic Republic of Congo
Source: Front Pain Res (Lausanne). 2024 Nov 22;5:1391666. doi: 10.3389/fpain.2024.1391666 (PMC11621210; doi:10.3389/fpain.2024.1391666)
Supplement: Supplementary file 11 [file Datasheet3.pdf]

frontiers

Supplementary Material

Article Title

Ange C M. Ngonde[1,2*], Philippe N. Lukanu[1,2], Ange Mubiala[3], Michel N. Aloniuthor[4]

[1]Polyclinique de Kinshasa, Kinshasa, The Democratic Republic of Congo

[2]Department de Médecine de Famille et soins de santé primaires, Université Protestante du Congo, Kinshasa, The Democratic Republic of Congo

[3]Institut National de Recherche Biomédicale (INRB), Kinshasa, The Democratic Republic of Congo

[4]Département de Pédiatrie, Cliniques Universitaires de Kinshasa, Faculté de Médecine, Université de Kinshasa, Kinshasa , The Democratic Republic of Congo

*** Correspondence:** Ange Christian MambakasaNgonde* angondemc@gmail.com

1 Supplementary Figures and Tables

For more information on Supplementary Material and for details on the different file types accepted, please see here.

1.1 Supplementary Figures

Table 1: Inclusion and exclusion criteria

Inclusion criteria	Exclusion criteria
-Diagnosed with major sickle cell syndrome (SCD) (SS, SC or Sbthal)<br><br>- Aged at least 2 years<br><br>- Availability of LDH assay values obtained outside of crisis episodes.<br><br>- To have at least two documented LDH assay values obtained during two differents follow-up periods : either before initiation of hydroxyurea (HU) treatment or during HU therapy.<br><br>- Availability of LDH assay values during concomitant use of hydroxyurea (HU) and arginine-containing supplement (ARG).	- LDH data collected during crisis episodes<br><br>- Cases without LDH values during the third follow-up period.<br><br>- Cases with LDH values available for only one follow-up period

Table 2: Patient's characteristics

**Patient's characteristics**	**Total**	**Patient Age group**	
		0 - 14 years	15+ years
**Sex**<br><br>**Female**	20.64 ± 8.38<br>11 (35.5)	6.6 ± 3.29<br>5 (45.5)	18.0 ± 4.65<br>6 (54.5)
**Male**	8.8 ± 3.56<br>20 (64.5)	9.53 ± 3.46<br>15 (75.0)	23.8 ± 11.21<br>5 (25.0)
**Patient weight**	20.64 ± 8.38	18.6 ± 6.2	40.4 ± 9.7
**LDH** Baseline	649.73 ± 347.28	632.81 ± 389.41	694.83 ± 219.70
**Total**	31	11 (35.5)	20 (64.5)
**Patient Age**			
Mean age& SD	13.00± 8.04		
Median& quatiles	12.00 (9.00 – 16.00)		
Minimum	2		
Maximum	43		

Table 3: LDH values among sickle cell patients during the three observation phases

**Patient's characteristics**	**Total**	**Age**		**P-value**
	**n=31**	**0 – 14 years**	**15 years and upper**	**Wilcoxon test**

		n=20	n=11	
**Sex**				
Male	13.10±8.68	9.53± 3.46	6.60±3.29	
	11.50 (41)	11 (12)	6 (7)	0.001
Female	12.82±7,11	18,0±4,65	23,80±12,21	
	15.0 (24)	16 (12)	19 (28)	
**LDH**				
Baseline	649.73 ± 347.28	632.81 ± 389.41	694.83 ± 219.70	0.001
%LDHnle	216.7%,			
phase 1	661.56 ± 367.39	622.20 ± 240.50	720.60 ± 513.33	0.001
%LDHnle	220.3%,			
phase 2	529.90 ± 346.3	500.13 ± 150.34	584.05 ± 558.40	0.001
%LDHnle	176.6%			
**Hb**				
Baseline	7.66±1.06	7.77±1.16	7.32±0.64	0.001
Phase 1	7.96 ± 1.33	7.81±1.39	8.18±1.27	0.578
Phase 2	7.71 ± 1.26	7.74±1.32	7.67±1.21	0.354
**Hct**				
Baseline	22.86 ± 3.09	23.17±3.27	22.08 ±2.69	0.001
Phase 1	23.74 ± 3.83	23.13±3.84	24.54±3.86	0.001
Phase 2	23.01 ± 3.24	22.86±2.60	23.25±4.22	0.001
**WBC**				
Baseline	12747.3± 3744	12762.5±3714.5	12706.7±4181	0.001
Phase 1	10983.3 ± 4297.5	11914.3±4402.6	9680.0±3995.5	0.001
Phase 2	11636.6 ± 3916	12515.8±4143.3	10118.2 ±3094.1	0.001

*LDH: Lactate dehydrogenase; Hb: Hemoglobin; Hct: Hematocrit; WBC: White blood cell*Normal LDH values: Female: 135 - 214 U/L; Male: 135 - 225 U/L; Children (2 to 15 years): 120 - 300 U/L; Newborns (4 to 20 days): 225 - 600 U/L.

*The difference between three phase is significant (p-value mann Whitney).

Table 4: LDH comparison in all 3 phases

	P-value	*Mean Difference*	*95 % confiance intervalle of the différence*

			lower	*Upper*
LDH_ baseline		649.75	495.75	803.70
LDH Phase 1	0.002**	661.56	509.9079	813.2121
LDH Phase 2		529.90	402.8788	656.9277
LDH Baseline	0.349	347,284	234.00	1907.00
LDH Phase 1		367,39228	193.00	1715.00
LDH Baseline	0.017*	649.727	495.75	803.70
LDH Phase 2		529.90323	402.8788	656.9277
LDH Phase 1	0.017*	649.727	495.75	803.70
LDH Phase 2		529.90323	402.8788	656.9277

**P value(*Friedman Test*)<0.05. With P=0,002, the difference in LDH in all 3 phases is statistically significant

*P value(*Wilcoxon Test*)<0.05, the difference in LDH between these 2 phases is statistically significant

Table 5: Correlation between LDH and other biological markers (Hb, Hct and WBC) during 3 phases

	Correlation Statistics	***Hb***	***Hct***	***WBC***
LDH baseline				
	Spearman Rho	-0.304*	-0.283*	0.274*
	P-value	0.008	0.015	0.017
LDH_ Phase1				
	Spearman Rho	-0.331^{ns}	-0.289^{ns}	0.396^{ns}
	P-value	0.142	0.203	0.068
LDH_ Phase2				
	Spearman Rho	-0.599*	-0.612*	0.406*
	P-value	.153	.006	0.903

*P<0.05

Supplementary Figure 1. The figure legends are required to have the same font as the main text, 12 point normal Times New Roman, single spaced. Please use a single paragraph for each legend and prepare the figures keeping in mind the PDF layout.
