# Supplementary material for: Arginine supplementation improves lactate dehydrogenase levels in steady-state sickle cell patients: preliminary findings from Kinshasa, the Democratic Republic of Congo
Source: Front Pain Res (Lausanne). 2024 Nov 22;5:1391666. doi: 10.3389/fpain.2024.1391666 (PMC11621210; doi:10.3389/fpain.2024.1391666)

frontiers

Schema of international longitudinal study of Arginine supplementation and its effect on improving LDH levels in steady-state sickle cell disease (SCD)patients

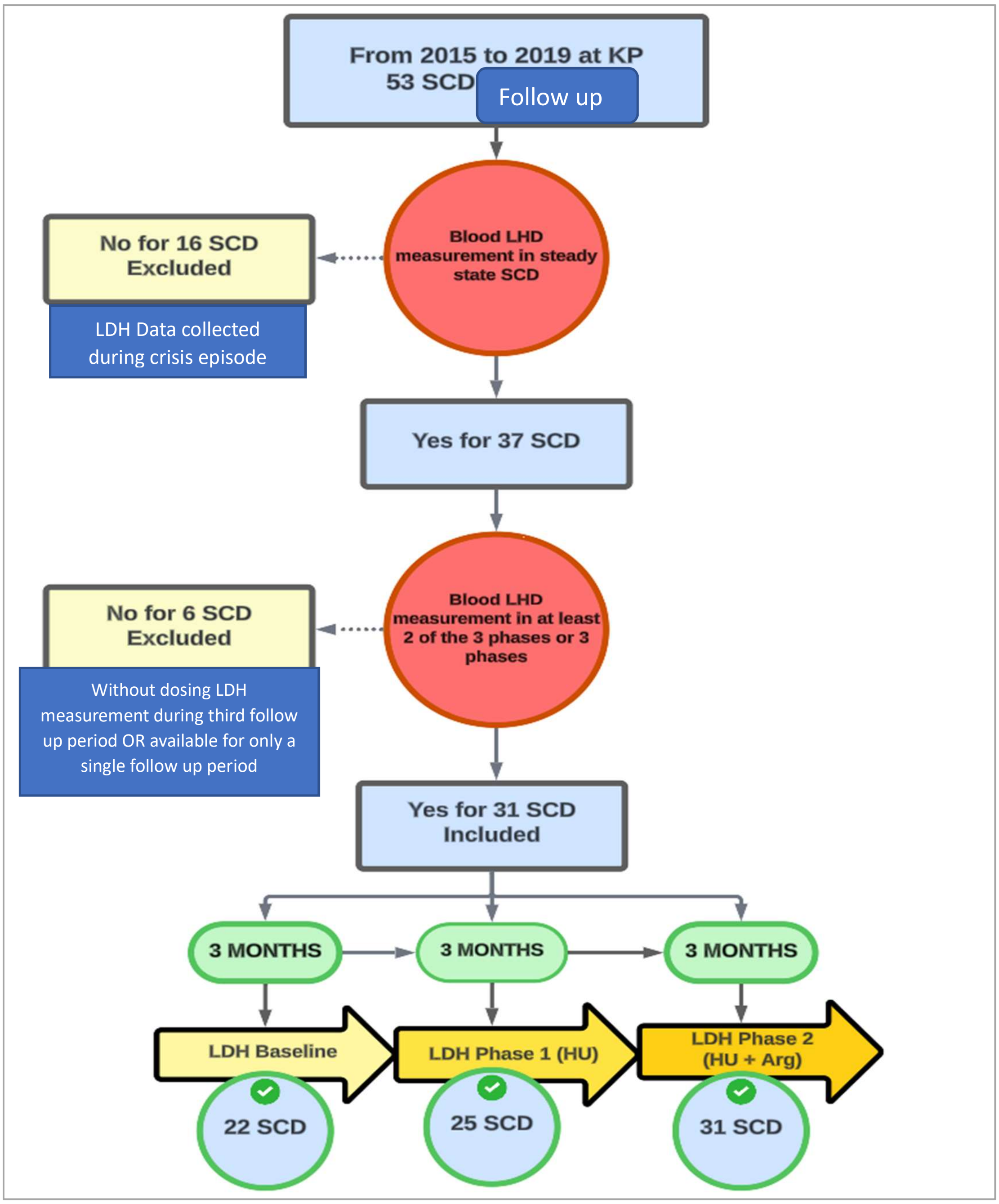

Supplement: Supplementary file 12 [file Datasheet4.pdf]
